# A prognostic signature for lung adenocarcinoma by five genes associated with chemotherapy in lung adenocarcinoma

**DOI:** 10.1111/crj.13723

**Published:** 2023-12-10

**Authors:** Xiaofeng Li, Chunwei Xu, Yonghua Min, Zhanqiang Zhai, Youcai Zhu

**Affiliations:** ^1^ Department of Thoracic Disease Diagnosis and Treatment Center Zhejiang Rongjun Hospital, The Third Affiliated Hospital of Jiaxing University Jiaxing China; ^2^ Institute of Cancer and Basic Medicine (ICBM) Chinese Academy of Sciences Hangzhou China

**Keywords:** chemotherapy, Gene Expression Omnibus (GEO), lung adenocarcinoma, risk score

## Abstract

**Background:**

Lung adenocarcinoma (LUAD) is one of the most common subtypes of lung cancer. Finding prognostic biomarkers is helpful in stratifying LUAD patients with different prognosis.

**Methods:**

We explored the correlation of LUAD prognosis and genes associated with chemotherapy in LUAD and obtained data of LUAD patients from the Cancer Genome Atlas (TCGA) and Gene Expression Omnibus (GEO) databases. Drug sensitivity data were acquired from the Genomics of Drug Sensitivity in Cancer (GDSC) database. Differential and enrichment analyses were used to screen the target genes utilizing limma and “clusterProfiler” packages. Then univariate and LASSO Cox analyses were used to select the prognosis‐related genes. Survival analysis was used to estimate the overall survival (OS) of different groups.

**Results:**

Twenty‐three differentially expressed genes (DEGs) were screened between LUAD samples and healthy samples, and *BTK*, *FGFR2*, *PIM2*, *CHEK1*, and *CDK1* were selected to construct a prognostic signature. The OS of patients in the high‐risk group (risk score higher than 0.69) was worse than that in the low‐risk group (risk score lower than 0.69).

**Conclusion:**

The risk score model constructed by five genes is a potential prognostic biomarker for LUAD patients.

## Graphical abstract

The prognostic signature in LUAD was constructed according to *BTK*, *FGFR2*, *PIM2*, *CHEK1*, and *CDK1*. The LUAD patients in high‐risk group exhibited a poor prognosis. The risk score model was a potential prognostic biomarker for LUAD patients.

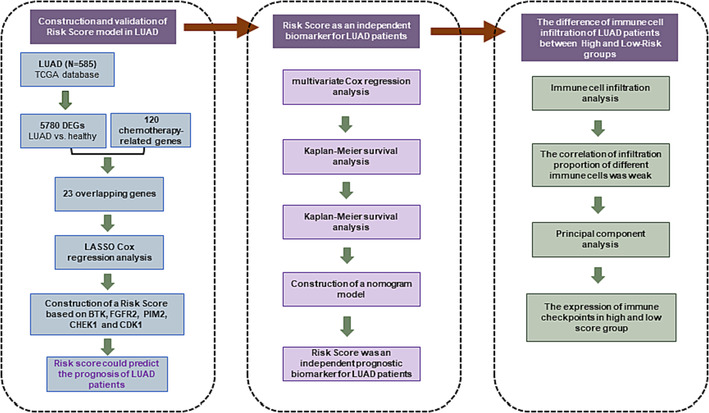

**Abbreviations**
DEGs: differentially expressed genes
GDSC: Genomics of Drug Sensitivity in Cancer
GEO: Gene Expression Omnibus
LUAD: lung adenocarcinoma
NSCLC: non‐small cell lung cancer
OSRs: overall survival rates
SCLC: small cell lung cancer
TCGA: The Cancer Genome Atlas

## 1 INTRODUCTION

According to the GLOBOCAN 2020, lung cancer, killing nearly 1.8 million people every year in the world, is the most frequent cause of cancer‐related deaths.^1^ In China, lung cancer has been the leading cancer type for many years, with an increasing of new cases form 0.3 million in 2015 to 0.42 million in 2020.^2^ Lung cancer can mainly be classified into non‐small cell lung cancer (NSCLC) and small cell lung cancer (SCLC). Lung adenocarcinoma (LUAD), the most common histological subtype of NSCLC, accounting for nearly 40% of all lung cancer cases.^3^ Smoking and second hand smoke have been main risk factors for lung cancer for many years, and other risk factors such as air pollution, environmental exposure, and genetic susceptibility also contribute to lung cancer.^4^ With the improvement of control and treatment options, 5‐year survival rates of LUAD diagnosed at early stage have remarkably increased.^5^, ^6^ But for patients with metastasis or late‐stage LUAD, the prognosis has not been improved substantially.^7^ Finding effective prognostic biomarkers may be helpful in stratifying patients with different prognosis and therefore in designing personalized treatment therapy.^8^ Moreover, due to the heterogeneity of cancer cells, more biomarkers are urgently required to explore.^9^ Due to the development of medical techniques, more molecular biomarkers for LUAD have been identified.

Many studies have identified prognostic signature for lung cancer patients through bioinformatics methods. For example, Gong et al. have constructed a risk score prognostic model for NSCLC patients based on the inflammatory‐related genes.^10^ Chemotherapy‐related genes have been reported to be associated with the prognosis of gastric adenocarcinoma and have been used to construct prognostic model.^11^ However, studies on the relationship between chemotherapy‐related genes and lung cancer have rarely been studied.

In this study, we downloaded data of LUAD patients from public databases to analyze and aimed to explore the relationship between the prognosis of LUAD and chemotherapy‐related genes in LUAD, hoping to construct a prognostic signature to stratify LUAD patients with different prognosis.

## 2 MATERIAL AND METHODS

### 2.1 Data collection

The mRNA expression data of 585 LUAD samples were obtained from The Cancer Genome Atlas (TCGA, https://tcga-data.nci.nih.gov/tcga/) database, comprised of 60 healthy samples and 525 cancer samples, including 501 LUAD samples with complete clinical information (Table 1). Besides, GSE68465 was downloaded from the Gene Expression Omnibus (GEO, https://www.ncbi.nlm.nih.gov/geo/) database. Samples were processed by Affymetrix Human Genome U133A Array.

**TABLE 1:** Clinicopathological characteristics of lung adenocarcinoma patients from TCGA‐LUAD database.

Characteristics		Patients (*N* = 501)	
		No.	%
Gender	Female	271	54.09%
	Male	230	45.91%
Age	≤66 (Median)	259	51.70%
	>66 (Median)	242	48.30%
Grade	I	269	53.69%
	II	119	23.75%
	III	80	15.97%
	IV	25	4.99%
	Unknown	8	1.60%
Survival time	Long (>5 years)	251	50.10%
	Short (<5 years)	52	10.38%
OS status	Dead	182	36.33%
	Alive	319	63.67%
Radiation	Yes	416	83.03%
	No	71	14.17%
	Unknown	14	2.79%
Tobacco	Yes	58	11.58%
	No	361	72.06%
	Unknown	82	16.37%

Drug sensitivity geneset of LUAD was also acquired from the Genomics of Drug Sensitivity in Cancer (GDSC, https://www.cancerrxgene.org/) database (Table S1). We selected 120 target genes which were associated with chemotherapy.

### 2.2 Differential expression analysis

We applied differential expression analysis on the research subjects based on limma package^12^ in R programming software (version 4.1.0, same below) to screen differentially expressed genes (DEGs) between the LUAD and healthy samples. The selection criteria included |Log_2_FC| > 1 and adjusted *p* value < 0.05.

### 2.3 Functional enrichment analysis

The Gene Ontology (GO) and Kyoto Encyclopedia of Genes and Genomes (KEGG) enrichment analyses were performed on the screened DEGs using “clusterProfiler” package^13^ in R programming software. Terms with adjusted *p* value < 0.05 were considered as significantly enriched.

### 2.4 LASSO Cox regression analysis

Univariate Cox regression analysis was applied on the LUAD samples based on the gene expression value. And *p* value < 0.05 was used as threshold to screen genes, which were significantly associated with the prognosis of LUAD. ESurv offers representative grouped variable selection techniques (lasso, elastic net, and Net), which can minimize the number of variables by taking into account their connections, and one of the most essential features of ESurv is the ability for users to do all survival analyses using their own data.^14^ LASSO was possibly the most extensively utilized penalization strategy for genomic data processing to date.^15^, ^16^, ^17^ Thus, we performed LASSO Cox regression using the R language “glmnet” package^18^ to further select the candidate prognosis‐related genes in LUAD. Moreover, the risk score of each LUAD sample was calculated using the candidate genes through the following formula:

$$\text{Risk Score}=\sum_{i=1}^{n}{\text{Coef}}_{i}\;*\;X_{i},$$

In this study, Coef_i_ and X_i_ were LASSO Cox coefficient and expression value of each gene, respectively. The patients were divided into low‐risk and high‐risk groups according to the median of risk score.

### 2.5 Survival analysis

Survival package and survminer package in R programming software were utilized to estimate the overall survival rates (OSRs) of high‐risk and low‐risk groups based on the Kaplan–Meier method. Log‐rank or Breslow tests were applied to examine the significance of difference of OSR between the high‐risk and low‐risk groups. A multivariate Cox regression model was used to analyze the independence of the risk score as a prognostic biomarker.

### 2.6 The proportion of immune cell infiltration

We used the CIBERSORT^19^ to calculate the proportion of 22 immune cells in each LUAD sample. The CIBERSORT software used the deconvolution algorithm to characterize the composition of immune cells utilizing the preset 547 barcode genes based on the gene expression matrix. The sum of the proportion of 22 immune cells in each LUAD patient was 1.

### 2.7 Construction of nomogram model

Nomogram model was widely used to predict the survival status of various cancers.^20^ The independent factors determined by multivariate Cox regression analysis were used to build a nomogram model based on the rms package of R programming software. A calibration curve of nomogram was used to compare estimated probability of nomogram model and actual probability.

### 2.8 Statistical analysis

We utilized the R programming software based on “K‐mean” method to classify mRNA expression data of LUAD patients with complete survival materials. ImmuneScore of LUAD patients was calculated by the “ESTIMATE” package.

## 3 RESULTS

### 3.1 Twenty‐three genes associated with LUAD carcinogenesis and chemotherapy

**FIGURE 1:**
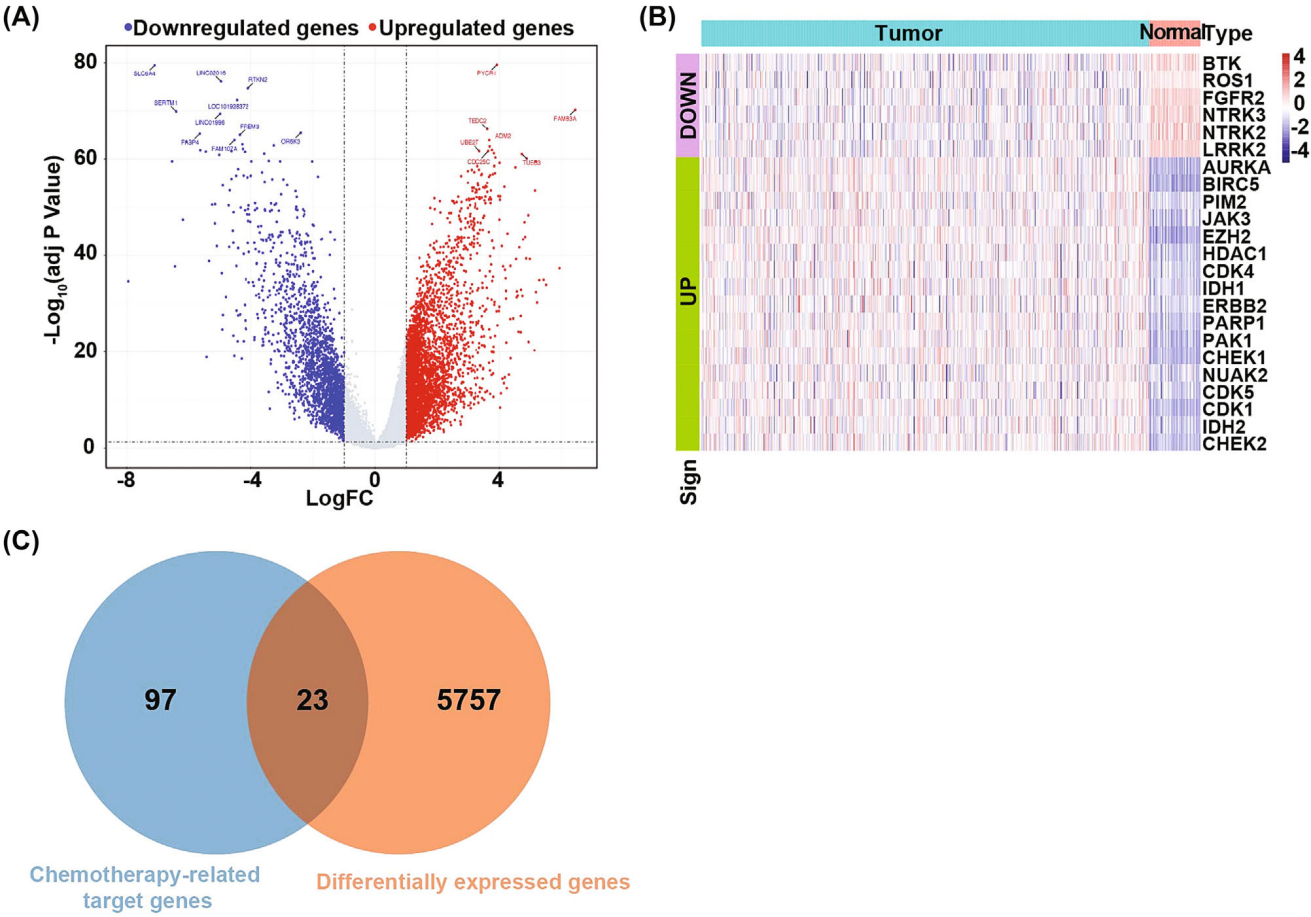
Results of differential expression analysis. (A) The volcano plots of differentially expressed genes (DEGs) between lung adenocarcinoma patients and healthy subjects. The horizontal and vertical axes represented the Log_2_FC and the −Log_10_ (FDR), respectively. The blue and red dots represented upregulation and downregulation genes respectively. (B) The hot map of DEGs. The horizontal axis was research subjects, and the vertical axis was types of genes. The red represented high expression of genes, and the blue represented low expression of genes. The green was upregulated genes and the purple was downregulated genes. (C) The Venn diagram of the overlap genes between the DEGs and drug sensitive genes.

We screened 5780 DEGs, including 3743 upregulated genes and 2037 downregulated genes (Figure 1A). Expression values of DEGs differed significantly between LUAD samples and healthy samples (Figure 1B).

Twenty‐three overlap genes were selected between the 5780 DEGs and the 120 chemotherapy‐related target genes (Figure 1C).

**FIGURE 2:**
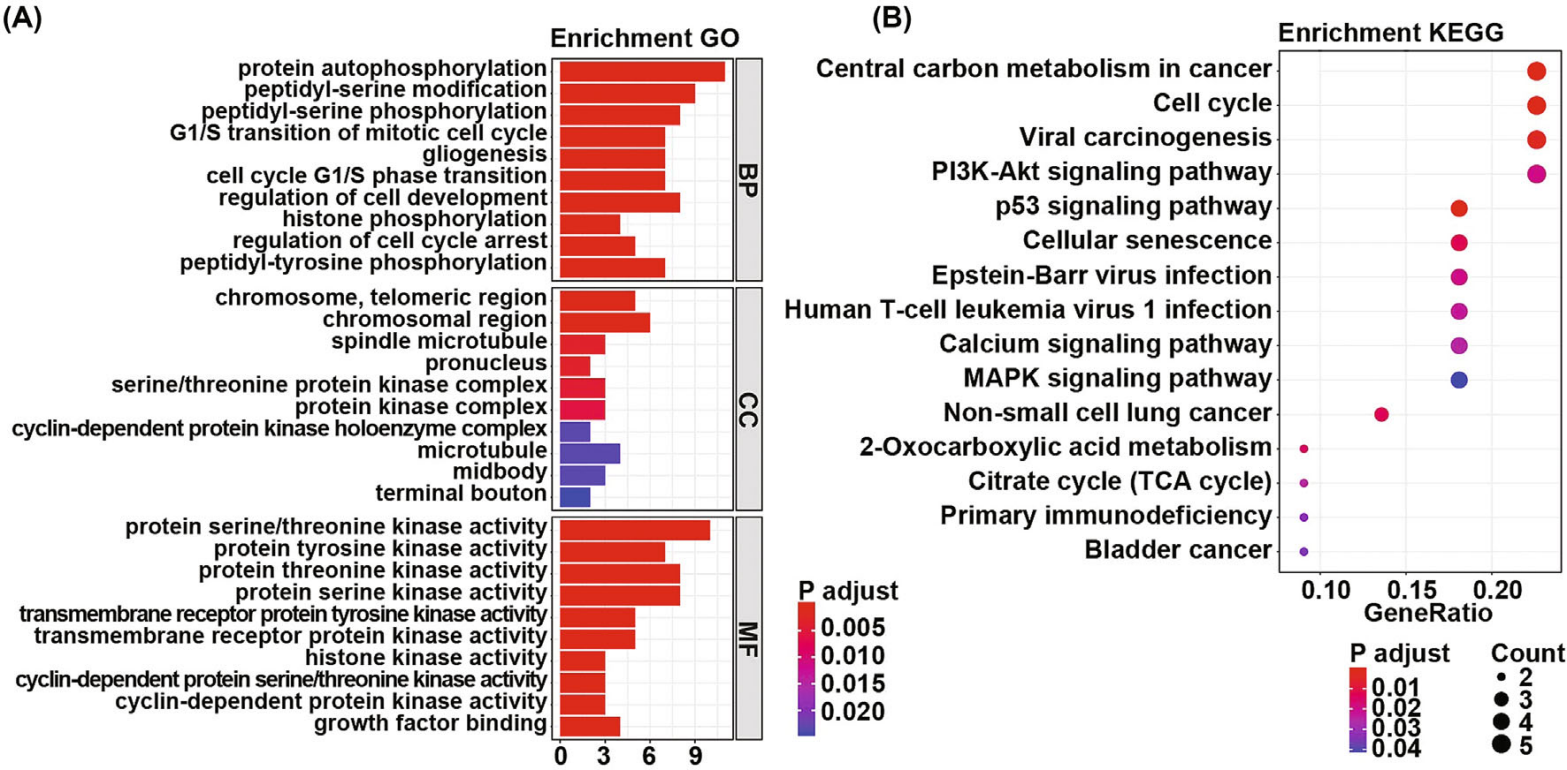
Results of enrichment analysis. (A) The significantly enriched GO terms. The horizontal axis was the number of enriched genes, and the vertical axis was the GO terms. (B) The significantly enriched KEGG pathways. The horizontal axis was the number of enriched genes, and the vertical axis was the KEGG pathways.

The enrichment analysis showed that these 23 overlapping genes were significantly enriched in 585 GO term such as the protein autophosphorylation, chromosome telomeric region, protein serine/threonine kinases activity, and 15 KEGG pathway, including central carbon metabolism in cancer and cell cycle. The most significantly enriched GO terms were listed in Figure 2A. The most significantly enriched KEGG pathways were listed in Figure 2B. The full results of GO and KEGG enrichment analyses were listed in Table S2.

### 3.2 Construction and validation of risk score model

**FIGURE 3:**
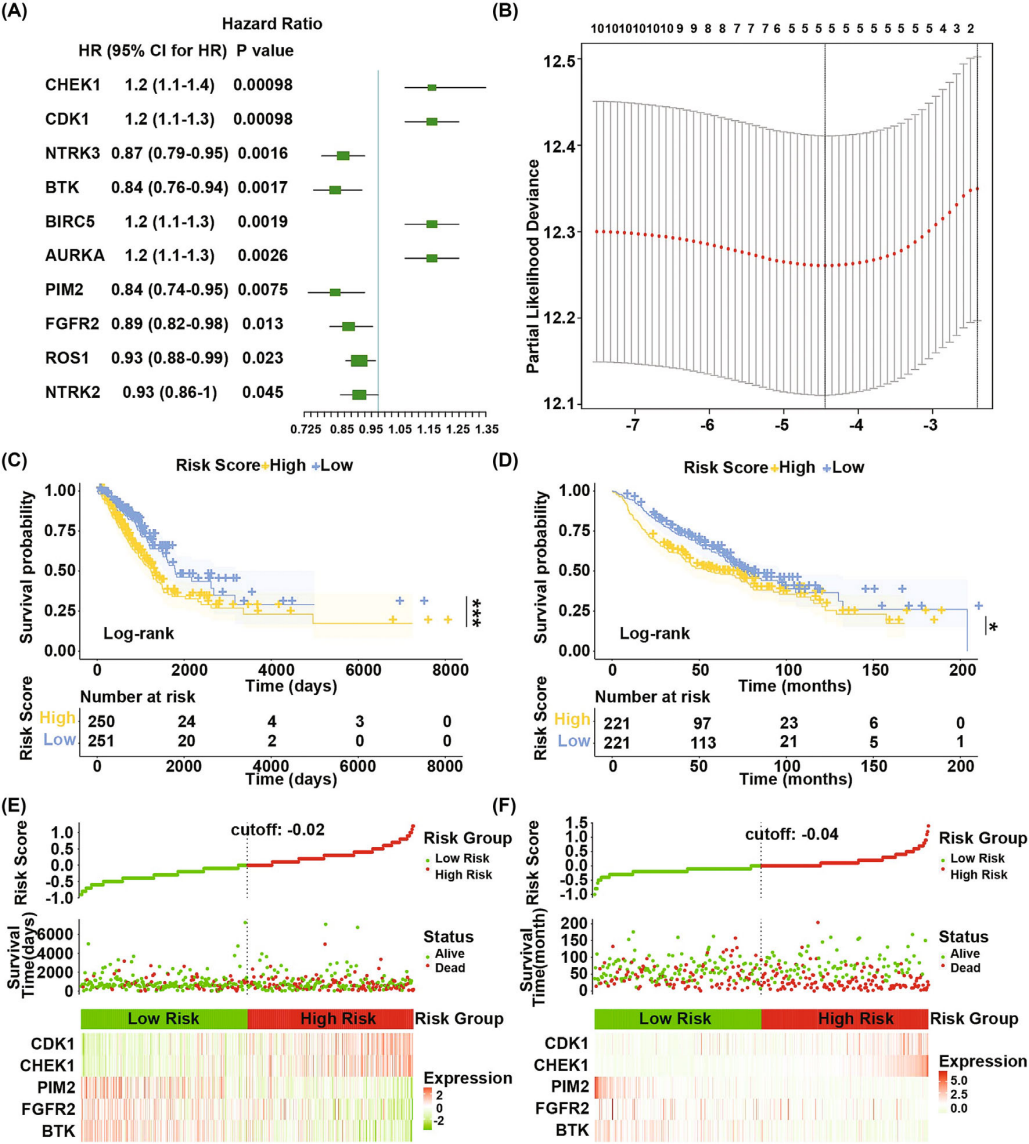
Construction of prognostic model for LUAD. (A) The forest plot of univariate Cox regression analysis to list top 10 genes, which were significantly related to the prognosis of LUAD. HR was short for Hazard ratio and 95%CI was 95% confidential interval. (B) The best tuning parameter lambda determined by LASSO Cox regression analysis. The horizontal axis was log (lambda) and the vertical axis was partial likelihood deviance the lowest of which corresponded to the best lambda. (C) The Kaplan–Meier survival curve of TCGA dataset. The horizontal and vertical axes were time and survival probability. The yellow represented high‐risk group, and the blue represented low‐risk group. *p* value was calculated by log‐rank test. (D) The Kaplan–Meier survival curve of GSE68465 dataset. The horizontal and vertical axes were time and survival probability. The yellow represented high‐risk group, and the blue represented low‐risk group. *p* value was calculated by log‐rank test. (E and F) The estimated risk score of each patient was sorted from the smallest to the largest. The vertical dotted line was the median of risk score. The distribution difference of survival time and hub gene expression between high‐risk group and low‐risk group was distinguished by median of risk score.

In TCGA dataset, hazard ratio of each gene was calculated by the univariate Cox regression analysis based on the 23 overlap genes as continuous variate. *p* value < 0.05 was used as selection criteria to screen the top 10 genes (Figure 3A). Then the 10 genes were optimized to five by LASSO Cox regression analysis (Figure 3B, the lowest lambda value). They were *BTK*, *FGFR2*, *PIM2*, *CHEK1*, and *CDK1*.

Then the gene expression values were multiplied by the LASSO Cox coefficient to construct prognostic risk score model as follows: Risk Score = (−0.06151767 × *BTK*) + (−0.05733390 × *FGFR2*) + (−0.14229387 × *PIM2*) + (0.10166837 × *CHEK1*) + (0.09975234 × *CDK1*). Patients in the TCGA and GEO databases were grouped into high‐risk and low‐risk groups according to the median of risk score. Survival analysis showed that overall survival (OS) of patients in the high‐risk group was worse than that in the low‐risk group in both TCGA dataset (*p* = 0.00073) (Figure 3C) and GEO dataset (*p* = 0.016) (Figure 3D).

In Figure 3E,F, green dots and red dots represented alive patients and dead patients. There were more dead patients in the high‐risk group compared to the low‐risk group.

These results suggested that risk score model constructed by *BTK*, *FGFR2*, *PIM2*, *CHEK1*, and *CDK1* was a reliable prognostic model.

### 3.3 Risk score as an independent biomarker for LUAD patients

**FIGURE 4:**
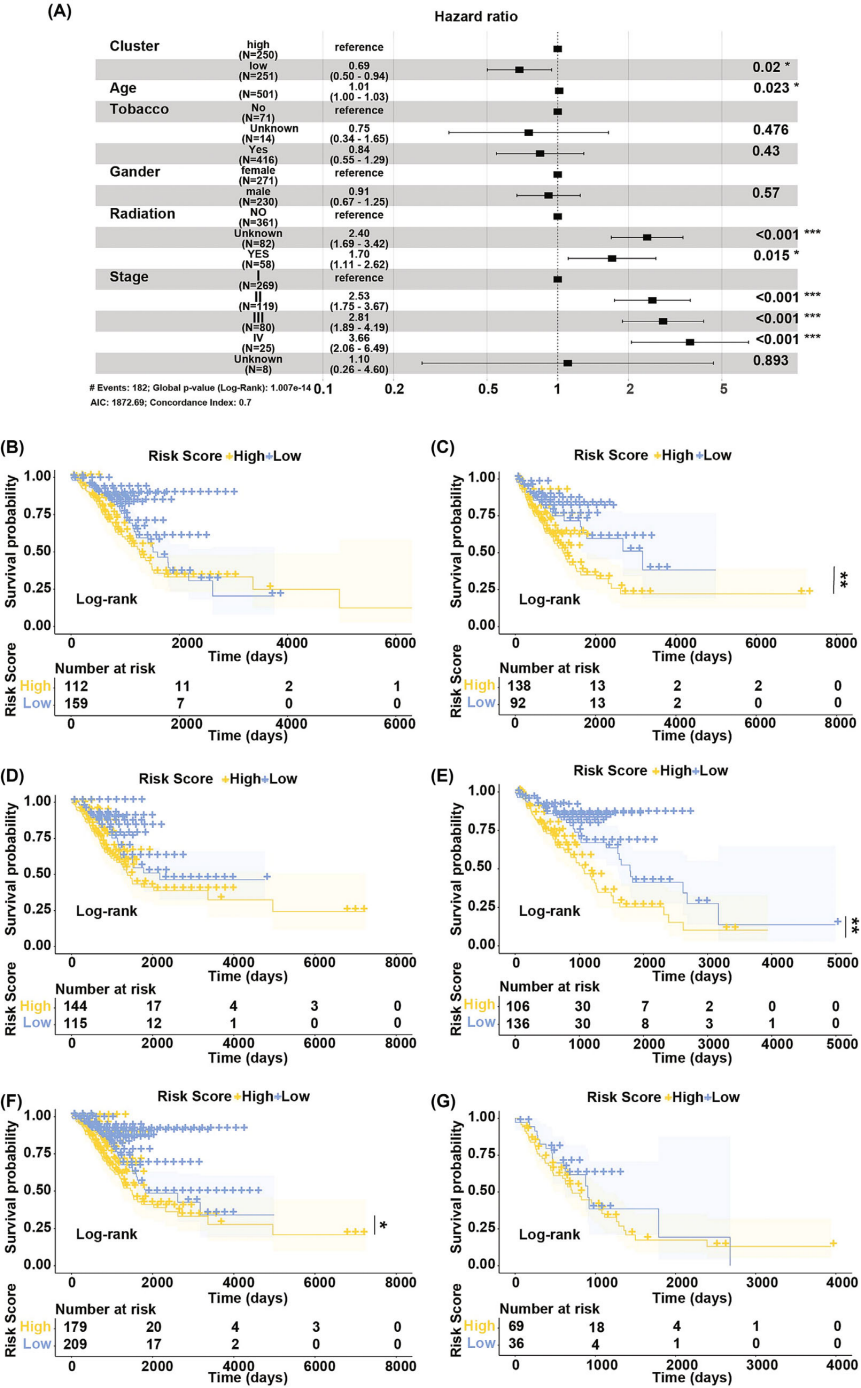
Risk score as an independent prognostic biomarker. (A) The forest plot of multivariate Cox regression analysis. Compared to the reference patients, patients with hazard ratio > 1 were with higher death risk and patients with hazard ratio < 1 were with lower death risk. (B and C) The Kaplan–Meier survival curve of female and male subgroups. (D and E) The Kaplan–Meier survival curve of younger than 66 years old and older than 66 years old subgroups. (F and G) The Kaplan–Meier survival curve of stage I/II and stage III/IV subgroups.

Six factors (comprising age, gender, stage, tobacco history, radiation, and median of risk score) were included in the multivariate Cox regression analysis. The results showed that the median of risk score, age, stage, and radiation were connected with the OS (Figure 4A). The patients with low‐risk score had lower death risk (HR = 0.69, 95%CI: 0.50–0.94, *p* = 0.02), and this suggested that risk score was a reliable prognostic biomarker.

The patients were then regrouped based on the age, gender, and stage (I/II, III/IV) to perform Kaplan–Meier survival analysis. The results showed that the OSR of patients in the high‐risk group was lower than that in the low‐risk group in the male subgroup (*p* = 0.0031) (Figure 4C), older than 66 years old subgroup (*p* = 0.0012) (Figure 4E), and stage I/II subgroup (*p* = 0.014) (Figure 4F). There no significant difference of OSRs between high‐risk and low‐risk groups in the female (Figure 4B), younger than 66 years old (Figure 4D), and stage III/IV (Figure 4G) subgroups. These indicated that the risk score was an independent prognostic biomarker for LUAD patients.

### 3.4 Nomogram model to predict the survival status of LUAD patients

**FIGURE 5:**
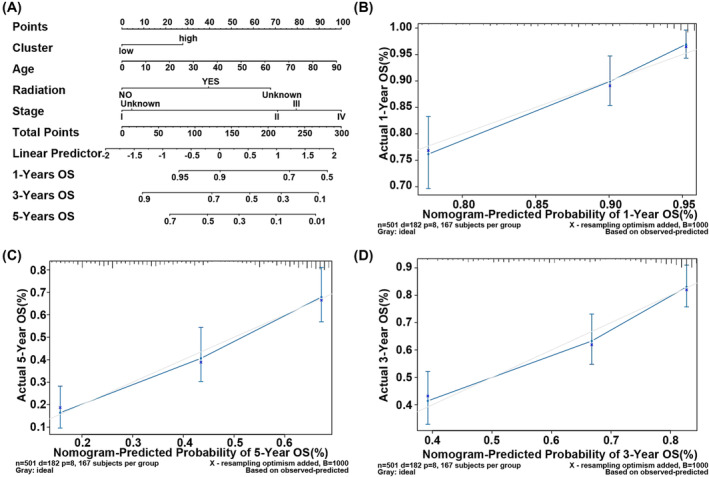
The survival status of LUAD patients predicted by nomogram model. (A) The nomogram model to predict survival of patients in 1 year, 3 years, and 5 years. (B–D) The calibration curve of nomogram model. The horizontal axis was the estimated probability of nomogram and the vertical axis was the actual survival probability.

A nomogram model was constructed by the independent prognostic factors identified by the multivariate Cox regression analysis (Figure 5A). The calibration curves were close to the ideal curve (a 45° line through origin with the slop 1) in Figure 5B–D, indicating that the estimation was in good accordance with the actual results.

### 3.5 The expression trend of the genes in different stages

**FIGURE 6:**
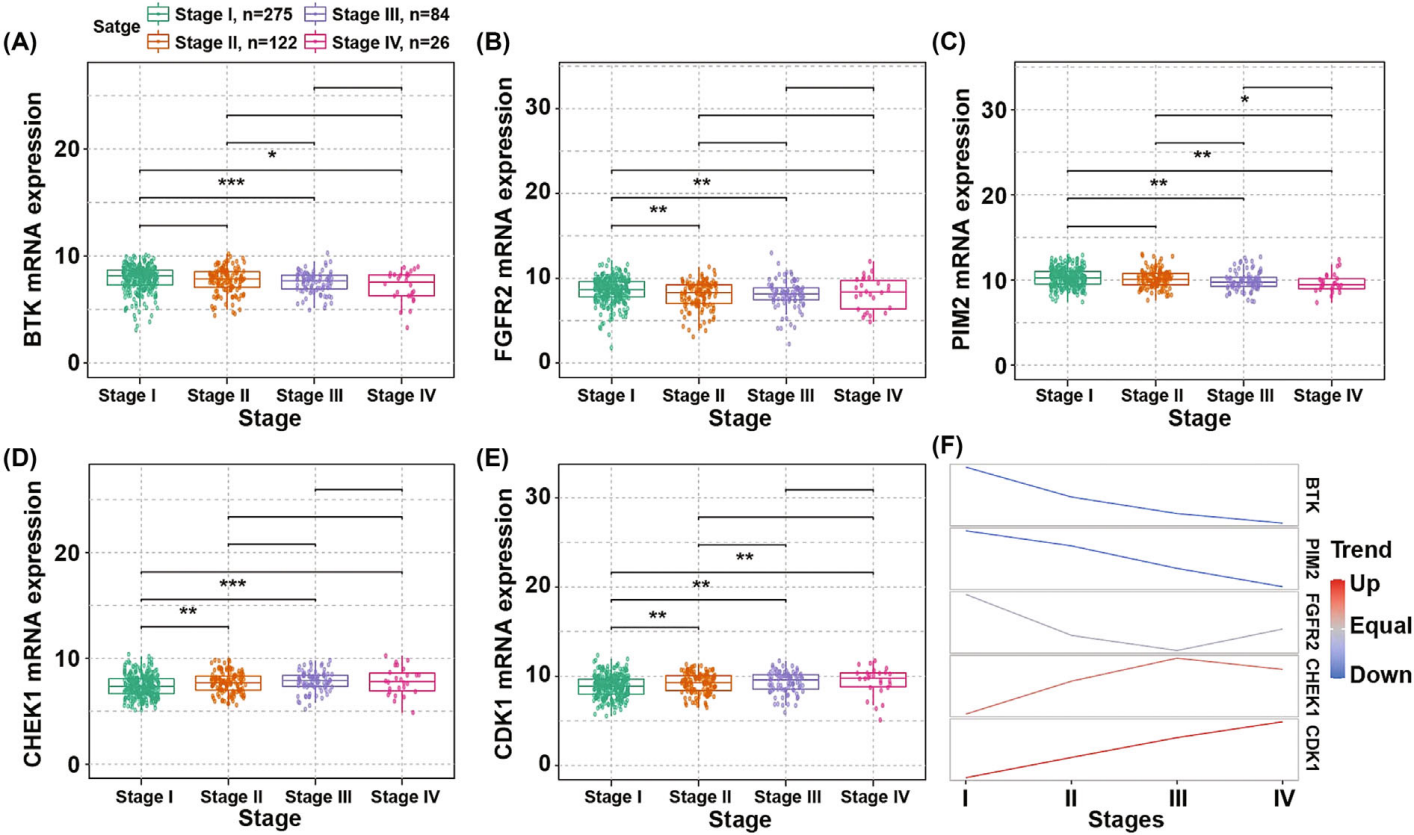
Expression trend in different stage. (A–E) The box plots of *BTK*, *FGFR2*, *PIM2*, *CHEK1*, and *CDK1* in different stages. (F) The *BTK*, *FGFR2*, *PIM2*, *CHEK1*, and *CDK1* expression trend in different pathologic stages of LUAD. The blue represented descending trend and the red represented ascending trend.

There were differences of *BTK*, *FGFR2*, *PIM2*, *CHEK1*, and *CDK1* expression in different stages. Compared to stage I, *BTK* and *PIM2* expressions were significantly decreased in stages III and IV, and the expression of *FGFR2* was remarkably decreased in stages II and III (Figure 6A–C). Moreover, compared to stage I, the expression of *CHEK1* was significantly increased in stages II and III, and *CDK1* was highly expressed in stages II, III, and IV (Figure 6D,E).

The expression of *BTK* and *PIM2* had descending trend in different stages, while the expression of *CDK1* had ascending trend in different stages. The trend of *FGFR2* and *CHEK1* expression turned at stage III (Figure 6F).

These suggested that the expression of *BTK*, *FGFR2*, *PIM2*, *CHEK1*, and *CDK1* differed significantly in different stages, which may assist treatment management in clinic.

### 3.6 The difference of immune cell infiltration of LUAD patients between the high‐risk and low‐risk groups

**FIGURE 7:**
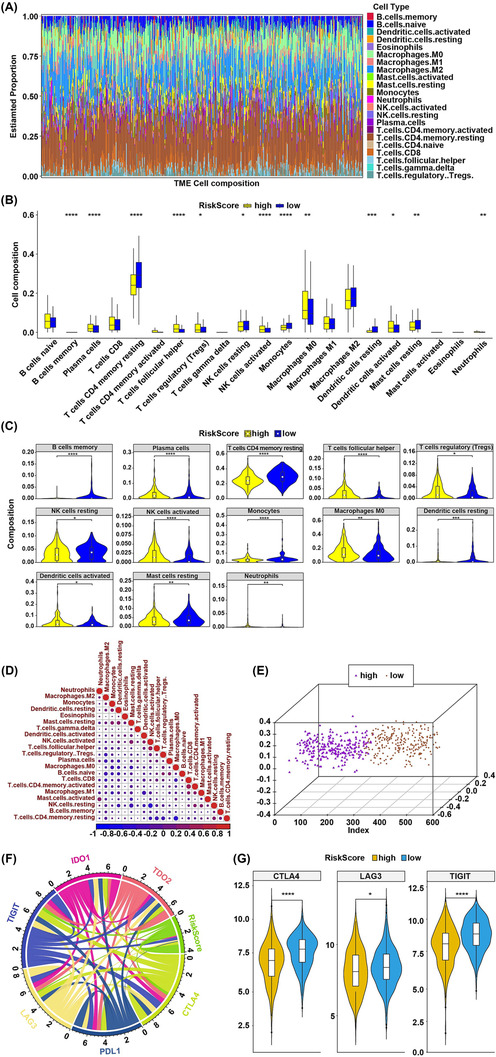
The immune cell infiltration of LUAD patients in the high‐risk and low‐risk groups. (A) The relative proportion of immune cell in all patients. (B) The violin diagram of differentially infiltrated immune cells between the high‐risk and low‐risk groups. The horizontal axis was the 21 immune cells, and the vertical axis was the relative proportion of immune cells. *p* value was calculated by Wilcoxon method (ns: *p* > 0.05, *: *p* ≤ 0.05, **: *p* ≤ 0.01, ***: *p* ≤ 0.001, ****: *p* ≤ 0.0001). (C) The violin diagram of significantly differentially infiltrated immune cells between the high‐risk and low‐risk groups. The horizontal axis was the high‐risk and low‐risk groups. The vertical axis was the relative proportion of immune cells. *p* values were calculated by Wilcoxon method (ns: *p* > 0.05, *: *p* ≤ 0.05, **: *p* ≤ 0.01, ***: *p* ≤ 0.001, ****: *p* ≤ 0.0001). (D) The correlation matrix of 21 immune cells. The red represented positive correlation, and the blue represented negative correlation. Darker color represented greater correlation. (E) The chord diagram of risk score and six immune checkpoints. The wider line represented greater correlation. (F) The violin diagram of significantly differentially expressed immune checkpoints between the high‐risk and low‐risk groups. The yellow and blue represented high‐risk and low‐risk groups, respectively. (G) The three‐dimensional diagram of PCA. The yellow represented low‐risk group, and the purple represented high‐risk group.

The immune cell infiltration results of 501 LUAD patients were shown in Figure 7A. The difference of infiltration of 21 immune cells (one result without infiltration was removed) in different patients represented the intrinsic characteristic. The infiltration proportion of 21 immune cells differed between the high‐risk and low‐risk groups (Figure 7B). The infiltration proportions memory of B cells, plasma cells, memory resting T cells, follicular helper T cells, regulatory T cells, resting NK cells, activated NK cells, monocytes, M0 macrophages, resting dendritic cells, activated dendritic cells, resting mast cells, and neutrophils differed significantly between high‐risk and low‐risk groups (Figure 7C). The infiltration proportions of plasma cells (*p* = 9.3e^−05^), regulatory T cells (*p* = 0.016), activated NK cells (*p* = 2.3e^−05^), M0 macrophages (*p* = 0.0019), and activated dendritic cells (*p* = 0.021) were higher in the high‐risk group compared to the low‐risk group. The correlation of infiltration proportion of different immune cells was weak (Figure 7D). The patients could be classified into two clusters based on the 13 significantly differed immune cells by PCA (Figure 7E).

The immune checkpoints' expression has been a biomarker for response to immune therapy. We analyzed the correlation between risk score and key immune checkpoints (*CTLA4*, *PDL1*, *LAG3*, *TIGIT*, *IDO1*, and *TDO2*) and found that risk score was related to all of them (Figure 7F). There was significant difference of *TIGIT*, *CTLA4*, and *LAG3* expression between the high‐risk and low‐risk groups (Figure 7G).

## 4 DISCUSSION

In this study, we constructed a novel five‐gene‐based prognostic signature to distinguish LUAD patients with different prognosis utilizing genes associated with chemotherapy and carcinogenesis of LUAD.

First, we screened the genes associated with LUAD carcinogenesis and chemotherapy using differential expression analysis. Then functional enrichment analysis was applied on the screened genes, and the results showed that the genes were enriched in the cancer‐related pathways such as cell cycle,^21^ PI3K‐Akt signaling,^22^ and non‐small cell lung cancer pathways. The screened genes were further optimized to five by the univariate Cox analysis and LASSO Cox regression analysis. A risk score model was built by *BTK*, *FGFR2*, *PIM2*, *CHEK1*, and *CDK1* to stratify the patients into high‐risk group and low‐risk group. The results of survival analysis showed that the prognosis of patients in the high‐risk group was worse than that in the low‐risk group in both TCGA and GEO datasets. Bi et al. reported that *BKT* is a potential prognostic factor for LUAD, indicating alterations in the tumor micro environment,^23^ and it was used to construct a prognostic signature for LUAD.^24^ The *PIM2* encodes a protooncogene that acts as a serine/threonine protein kinase to promote cell survival and participate in the progress of lung cancer.^25^ The *CHEK1* is related to poor prognosis of LUAD patients and is a potential biomarker for LUAD patients.^26^ Overexpression of *FGFR2* promotes progression and drug resistance of LUAD by suppressing activity of the receptor.^27^ Increased expression of *CDK1* in cancer patients is connected with higher risk of recurrence,^28^ and it is a promising prognostic marker for LUAD.^29^ These findings are in accordance with our results, suggesting that the risk score model, constructed by the screened genes, is a reliable prognostic signature for LUAD patients. Besides, our results are similar to those of other studies, in which abnormal expressions of the screened genes are also found in various cancers.^30^, ^31^, ^32^, ^33^ These suggested that the risk score model constructed by the screened genes is reliable to distinguish LUAD patients with different prognosis.

Sun and colleagues constructed a hypoxia‐related gene signature that could discriminate high‐ and low‐risk LUAD patients at an early stage.^34^ Nevertheless, whether model can be used to predict immune cell infiltration and response to immunotherapy in LUAD patients was unknown. Previous studies have reported that the prognostic signature structured using immune‐related genes or mitochondrial electron transport chain‐related genes could predict the prognosis of LUAD patients,^35^, ^36^ while these studies did not analyze the correlation between prognostic signature and key immune checkpoints. The increased infiltration proportion of regulatory T cells, activated NK cells, and activated dendritic cells may be connected with a poor prognosis in cancer patients by suppressing anti‐tumor immune response.^37^ Previous studies have reported that the infiltration of immune cells within malignant cells was closely related to the expression of immune checkpoints.^38^, ^39^ In this study, LUAD patients in the high risk exhibited a higher relative proportion of regulatory T cells, activated NK cells, and activated dendritic cells along with lower expression of CTLA4, LAG3, and TIGIT, and a poor prognosis, suggesting that the immunosuppressive environment and increased immune checkpoint expressions might be responsible for the inferior prognosis. This finding indicated that treatment with immune checkpoint inhibitors might benefit the low‐risk LUAD patients more than the high‐risk LUAD patients, resulting in a better outcome. Thus, the advantage of our prognostic model was that the risk score was connected to the immunosuppressive environment and immune checkpoint expression, assisting clinicians in selecting customized immunotherapy for LUAD patients.

The expression of *BTK* and *PIM2* was in descending trend from stage I to stage IV, while the expression of *CDK1* has an ascending trend from stage I to stage IV. The expression of *FGFR2* and *CHEK1* turned at stage III. The expression of the five genes differed significantly in different stages of LUAD. These may assist in distinguishing stages of LUAD patients and designing individual‐specific therapy. *CDK1* is reported to be highly expressed in late‐stage gastrointestinal stromal tumors but not in early‐stage gastrointestinal stromal tumors.^40^ *FGFR2* expression is also related to the stage in lung cancer and idiopathic pulmonary fibrosis.^41^ Drugs targeting *BTK* may act as B cell receptor signaling pathway inhibitors to be involved in chronic lymphocytic leukemia regime by regulating B cell differentiation.^42^, ^43^ These findings are in line with our results.

In summary, the risk score model we built is reliable to stratify LUAD patients with different prognosis. Furthermore, more clinical studies are needed to further verify the accuracy of the prognostic signature and to promote its clinical utility in individualized treatment management for LUAD patients.

## 5 CONCLUSION

Five genes associated with LUAD carcinogenesis and chemotherapy have been selected to construct a risk score model for predicting the prognosis of LUAD patients. And it is a potential independent prognostic biomarker for LUAD patients.

## AUTHOR CONTRIBUTIONS

Xiaofeng Li contributed to the design of the study and data analysis. Chunwei Xu, Yonghua Min, and Zhanqiang Zhai analyzed and interpreted the patient data. Xiaofeng Li collected and assembled data. Youcai Zhu drafted the manuscript. All authors reviewed and revised the original article. All authors read and approved the final manuscript.

## CONFLICT OF INTEREST STATEMENT

The authors declare that they have no competing interests.

## ETHICS STATEMENT

The ethical approval and informed consent were unnecessary as this study did not involve human subjects.

## Supporting information


**Table S1.** The drug sensitivity geneset in GDSC database.

**Table S2.** The full result of GO terms and KEGG pathways.

## Data Availability

The datasets analyzed during the current study are available in the Cancer Genome Atlas (TCGA, https://tcga-data.nci.nih.gov/tcga/) database, the Gene Expression Omnibus (GEO, https://www.ncbi.nlm.nih.gov/geo/, accession number GSE68465) database, and the Genomics of Drug Sensitivity in Cancer (GDSC, https://www.cancerrxgene.org/) database.
